# Incidence and predictors of pulmonary aspergillosis in patients with lung cancer: a systematic review and meta-analysis

**DOI:** 10.3389/fmed.2025.1560288

**Published:** 2025-04-28

**Authors:** Geling Teng, Feng Jin, Hua Zhang, Min Zhang

**Affiliations:** ^1^Department of Respiratory and Critical Care Medicine, Shandong Public Health Clinical Center, Jinan, China; ^2^Department of Chest Surgery, Shandong Public Health Clinical Center, Jinan, China; ^3^Department of Respiratory and Critical Care Medicine, Shandong Provincial Hospital Affiliated to Shandong First Medical University, Jinan, China

**Keywords:** incidence, predictors, pulmonary aspergillosis, lung cancer, systematic review, meta-analysis

## Abstract

**Background:**

Pulmonary aspergillosis is a rare but serious complication following lung cancer surgery, increasing the risk of mortality. The incidence of pulmonary aspergillosis and its risk factors among lung cancer patients is unknown. This study systematically investigates the incidence and associated risk factors of pulmonary aspergillosis in lung cancer patients.

**Methods:**

The databases PubMed, Web of Science, Scopus, Embase, CINAHL, and the Cochrane Library were comprehensively searched from their inception to March 2025. The overall incidence of pulmonary aspergillosis among lung cancer patients was analyzed using a random-effects model with logit transformation. Risk factors for pulmonary aspergillosis in lung cancer patients were presented as odds ratios (ORs) with 95% confidence intervals (CIs), calculated using a random-effects model.

**Results:**

Nine retrospective studies involving 20,138 patients with lung cancer were selected for the final analysis. The overall incidence of pulmonary aspergillosis in lung cancer patients was 2.4% (95% confidence interval [CI]: 1.5–3.2%). Subgroup analyses revealed higher incidences of pulmonary aspergillosis than corresponding subgroups in the following categories: Asia (2.8%; 95% CI: 2.0–3.7%), diagnosis by serological test (11.7%; 95% CI: 8.0–15.4%), patients with both non-small cell lung cancer and small cell lung cancers (3.6%; 95% CI: 2.0–5.2%), patients treated with chemoradiotherapy (5.7%; 95% CI: 1.6–9.7%), and pooled studies with moderate quality (2.9%; 95% CI: 1.7–3.2%). Moreover, the risk factors for pulmonary aspergillosis in lung cancer patients included male sex (OR: 1.96; *p* = 0.008), current or past smoking (odds ratio [OR]: 2.92; *p* < 0.001), chronic obstructive pulmonary disease (OR: 1.88; *p* = 0.011), interstitial lung disease (OR: 3.71; *p* < 0.001), pulmonary tuberculosis (OR: 2.79; *p* = 0.028), and treatment with double lobectomy (OR: 2.74; *p* < 0.001).

**Conclusion:**

Our study highlights pulmonary aspergillosis as a significant complication in lung cancer patients, with an overall incidence of 2.4%. The identified risk factors provide crucial insights for targeted screening and intervention in this patient population. Future research should focus on validating these findings in prospective studies and exploring the underlying biological mechanisms to develop more effective preventive and treatment strategies.

**Systematic review registration:**

This study was registered in the International Platform of Registered Systematic Review and Meta-analysis Protocols (INPLASY) platform (number: INPLASY2024100066).

## 1 Introduction

Lung cancer is the leading cause of cancer-related deaths globally, with over a million fatalities annually (1). Based on tissue characteristics, it can be categorized into small cell lung cancer (SCLC) and non-small cell lung cancer (NSCLC) (2). The development of lung cancer is a complex, multifaceted process involving numerous risk factors (3). Currently, the primary treatment approaches for lung cancer encompass surgical resection, chemotherapy, radiation therapy, and biological therapies (4). Nonetheless, despite the use of these multimodal strategies, a high rate of treatment failure and disease recurrence remain crucial factors contributing to poor patient outcomes (5).

Pulmonary aspergillosis is a severe yet frequently overlooked fungal lung infection, with an estimated global patient population exceeding three million; however, the epidemiological data concerning pulmonary aspergillosis remain inadequate (6). Currently, clinicians’ understanding of the risk factors contributing to the progression of pulmonary aspergillosis is often limited and incomplete (7). Pulmonary aspergillosis predominantly affects individuals with preexisting underlying respiratory disease, particularly those with tuberculosis (TB) (8, 9). A history of lung cancer treatment or previous thoracic surgery has been reported as a potential trigger for the development of pulmonary aspergillosis (10). Due to its relatively low incidence rate, current longitudinal studies on lung cancer patients provide limited data regarding the progression of pulmonary aspergillosis and its associated risk factors. The existing literature has reported variable incidences and inconsistent risk factors for pulmonary aspergillosis in lung cancer patients, mainly due to differences in study design, patient populations, and diagnostic criteria. Our study aims to bridge these knowledge gaps by conducting a comprehensive systematic review and meta-analysis. By pooling data from multiple studies, we aim to provide a more accurate estimate of the incidence of pulmonary aspergillosis in lung cancer patients. In addition, identifying consistent risk factors will enable clinicians to better stratify patients at high risk, facilitating targeted preventive strategies and early interventions. This, in turn, has the potential to improve patient outcomes, reduce healthcare costs associated with the management of this fungal infection, and contribute to a deeper understanding of the complex relationship between lung cancer and pulmonary aspergillosis. Thus, our research holds great promise for enhancing clinical practice and future research in this area.

## 2 Methods

### 2.1 Data sources, search strategy, and selection criteria

This review adheres to the requirements and reporting guidelines outlined in the Preferred Reporting Items for Systematic Reviews and Meta-Analyses (PRISMA) statement (11). This study was registered in the International Platform of Registered Systematic Review and Meta-analysis Protocols (INPLASY) platform (number: INPLASY2024100066). Studies reporting the incidence and risk factors associated with pulmonary aspergillosis in lung cancer patients were included. No restrictions were applied regarding language or publication status. PubMed, Web of Science, Scopus, Embase, CINAHL, and the Cochrane Library were searched for literature up to 26 March 2025, using the search terms ‘lung cancer’ and ‘Pulmonary aspergillosis’ [PubMed: (“lung cancer”[MeSH Terms] OR “lung cancer”[All Fields]) AND (“Pulmonary aspergillosis”[MeSH Terms] OR “Pulmonary aspergillosis”[All Fields]); Web of Science: “TS = [‘lung cancer’ AND ‘Pulmonary aspergillosis’ AND (‘incidence’ OR ‘risk factors’)]”; Scopus: “TITLE-ABS-KEY [‘lung cancer’ AND ‘Pulmonary aspergillosis’ AND (‘incidence’ OR ‘risk factors’)]”; Embase: (“lung cancer”: ab,ti OR “lung neoplasm”: ab,ti) AND (“Pulmonary aspergillosis”: ab,ti); CINAHL: “[‘lung cancer’ AND ‘Pulmonary aspergillosis’ AND (‘incidence’ OR ‘risk factors’)]”; Cochrane Library: (“lung cancer” AND “Pulmonary aspergillosis”)]. Furthermore, we manually examined the reference lists of all relevant original studies and reviews to identify and include any studies that met our inclusion criteria but may have been missed during the screening process, ensuring the comprehensiveness of our search.

The literature search and study selection were independently performed by two reviewers following a standardized procedure, and disagreements between reviewers were resolved through group discussion until a consensus was reached. The inclusion criteria are as follows: (1) Patients: all lung cancer patients, irrespective of cancer type or stage; (2) Exposure: patients infected with pulmonary aspergillosis; (3) Control: patients not infected with pulmonary aspergillosis; (4) Outcomes: the incidence of pulmonary aspergillosis or its predictors for pulmonary aspergillosis in lung cancer patients; and (5) Study design: prospective and retrospective observational studies.

### 2.2 Data collection and quality assessment

Two reviewers independently extracted the following information from the included studies: first author’s surname, publication year, country, study design, sample size, age, male proportion, body mass index (BMI), smoking proportion, diagnostic criteria for pulmonary aspergillosis, disease status, treatments, and investigated effect estimates. Then, these two reviewers independently assessed the quality of the included studies using the Newcastle–Ottawa Scale (NOS), which primarily comprises sections on selection (four items), comparability (one item), and outcome (three items) (12). Discrepancies in the results between reviewers regarding data collection and quality assessment were resolved by an additional reviewer referring to the original article.

### 2.3 Statistical analysis

The overall incidence of pulmonary aspergillosis in lung cancer patients was pooled using a random-effects model with logit transformation, and all models were fitted using restricted maximum likelihood estimation. For studies with zero events, a continuity correction of 0.5 was applied (13). Then, the predictors for pulmonary aspergillosis in lung cancer patients were assigned as odds ratios (ORs) with 95% confidence intervals (CIs), and the pooled analyses were calculated using the random-effects model (13, 14). Heterogeneity across included studies was assessed using *I*^2^ and Q-statistic tests, and significant heterogeneity was defined as *I*^2^ > 50.0% or *p* < 0.10 (15, 16). Sensitivity analyses were performed to assess the robustness of the pooled conclusion by sequentially removing individual studies (17). Subgroup analyses for the incidence of pulmonary aspergillosis in lung cancer patients according to country, diagnostic criteria, lung cancer, treatments, and study quality, and the differences between subgroups were compared using an interaction *t*-test, assuming the data met normal distribution (18). Publication bias was assessed using both qualitative and quantitative methods, including funnel plots, Egger’s test, and Begg’s test (19, 20). All reported *p*-values were derived from two-tailed tests, with combined results having a *p*-value < 0.05 considered statistically significant. The data analysis was conducted using STATA software, version 12.0 (StataCorp, College Station, TX, USA). All STATA commands used are shown in the Supplementary material.

## 3 Results

### 3.1 Literature search

**Figure 1:**
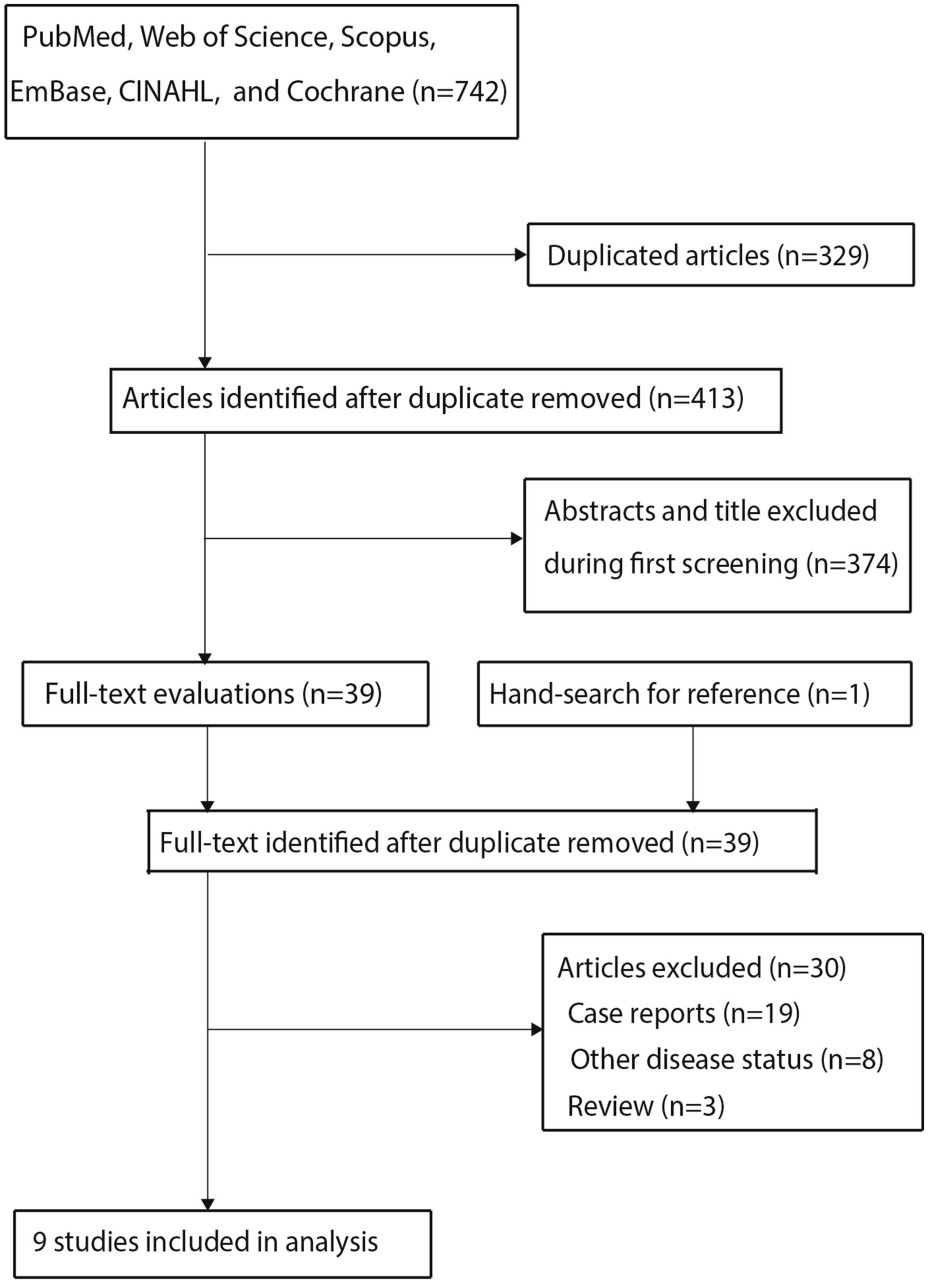
Preferred Reporting Items for Systematic Reviews and Meta-Analyses (PRISMA) flowchart regarding the study selection process.

A total of 742 articles were identified through electronic searches; all the retrieved articles were imported into a reference management software. The software’s duplicate-detection function was used to identify and remove exact duplicates based on title, author list, and publication details, resulting in the retention of 413 studies after removing the duplicate records. Then, 374 articles were excluded during title and abstract screening because they reported irrelevant topics. The remaining 39 studies were retrieved for full-text evaluation, of which 30 were excluded for the following reasons: case reports (*n* = 19), other disease statuses (*n* = 8), and reviews (*n* = 3). Reviewing the reference lists of relevant articles did not yield any new eligible studies. The remaining nine studies were selected for the final meta-analysis (21–29), and the details of the literature search and study selection process are shown in Figure 1.

### 3.2 Study characteristics

The baseline characteristics of the included studies and involved patients are summarized in Table 1. A total of 20,138 patients with lung cancer were identified from the nine studies, and the sample size ranged from 187 to 6,777. All included studies were retrospective cohorts. Two studies were conducted in Europe, and the remaining seven studies were conducted in Asia. Four of the included studies included both NSCLC and SCLC, while the remaining five studies included patients with NSCLC. Study quality was assessed using the NOS scale; four studies received seven stars, three studies received six stars, and the remaining two studies received five stars (Supplementary Table S1).

**Table 1:** Baseline characteristics of the included studies and the patients involved.

Study	Country	Study design	Sample size	Age (years)	Male (%)	BMI (kg/m^2^)	Smoking (%)	Diagnostic criteria for pulmonary aspergillosis	Disease status	Treatment	Number of pulmonary aspergillosis	Study quality
Yan et al. (21)	China	Retrospective	1711	61.0	72.2	NA	NA	EORTC/MSG. Proven IPA required histopathologic or microbiologic documentation of infection from tissues obtained by biopsy or in culture samples from a normally sterile site. Probable disease required (1) a host factors, including a recent history of neutropenia, corticosteroids use, or treatment with T-cell immunosuppressants; and (2) clinical features, including the presence of a new infiltrate on a chest computed tomography scan, such as dense lesions, air crescent signs, or a cavity; and (3) mycologic evidence	NSCLC and SCLC	NA	45	6
Tamura et al. (22)	Japan	Retrospective	475	68.0	68.0	NA	65.0	(i) Chronic pulmonary or systemic symptoms; (ii) cavitary pulmonary lesion(s) with evidence of paracavitary infiltrates; (iii) either a positive serum *Aspergillus* precipitin test result or isolation of *Aspergillus* spp.; (iv) elevated levels of inflammatory markers; (v) exclusion of other pulmonary pathogens; (vi) not immunocompromised	NSCLC	Lung resection	17	7
Sugimoto et al. (23)	Japan	Retrospective	187	NA	NA	NA	NA	Clinical, laboratory, and radiographic findings, including testing for 1,3-beta-D-glucan and galactomannan antigen, cultures, bronchoscopy, chest X-ray, and chest computed tomography	NSCLC	Chemoradiotherapy	6	5
Shin et al. (24)	Korea	Retrospective	3,423	63.0	63.6	23.8	29.8	(1) The presence of compatible clinical symptoms; (2) serological or microbiological evidence of *Aspergillus* infection, including positive serum *Aspergillus* precipitin tests (*Aspergillus fumigatus* Immunoglobulin G Enzyme-linked Immunosorbent Assay kit; IBL International, Hamburg, Germany) and isolation of *Aspergillus* spp. from respiratory specimens, or histological confirmation; (3) radiological findings compatible with evidence of disease progression; and (4) exclusion of alternative diagnoses, according to the widely accepted diagnostic criteria proposed by EORTC/MSG	NSCLC	Lung resection	56	6
Rønberg et al. (25)	Denmark	Retrospective	978	70.0	54.4	24.8	81.0	One or more cavities with or without a fungal ball present or nodules on thoracic imaging; Any direct or indirect mycological evidence from respiratory samples or from blood of *Aspergillus* spp. Infection; Exclusion of an alternative diagnosis; Disease present for at least 3 months.	NSCLC and SCLC	NA	16	5
Kim et al. (26)	Korea	Retrospective	6,777	63.0	61.3	23.9	26.6	European Society for Clinical Microbiology and Infectious Diseases/European Respiratory Society criteria: (1) Compatible clinical symptoms; (2) serological or microbiological evidence: positive serum *Aspergillus* precipitin test (*Aspergillus fumigatus* IgG ELISA kit; IBL International, Hamburg, Germany); isolation of *Aspergillus* species from a respiratory specimen, or histologic confirmation; (3) compatible radiological findings with overt progression; and (4) exclusion of alternative diagnosis	NSCLC	Lung resection	93	7
Choi et al. (27)	Korea	Retrospective	1872	63.0	78.3	23.4	30.8	Compatible symptoms, direct or indirect microbiological evidence of *Aspergillus* species, and compatible radiological findings	NSCLC and SCLC	Radiotherapy	59	7
Kuo et al. (28)	China	Retrospective	290	66.5	70.3	NA	NA	2021 EORTC/MSGERC Consensus Definitions of Invasive Fungal Diseases: Patients with a serum GM ODI ⩾ 1.0, BAL GM ODI ⩾ 1.0, or a combination of serum GM ODI ⩾ 0.7 and BAL GM ODI ⩾ 0.8 were diagnosed with IPA	NSCLC and SCLC	Chemotherapy	34	6
Whittaker et al. (29)	UK	Retrospective	4,425	66.7	48.3	26.9	73.2	(1) Progressive cavitary changes on a chest CT scan; (2) a positive sputum culture or positive *Aspergillus* IgG; and (3) the exclusion of alternative diagnoses.	NSCLC	Lung resection	11	7

### 3.3 Incidence of pulmonary aspergillosis in patients with lung cancer

**Figure 2:**
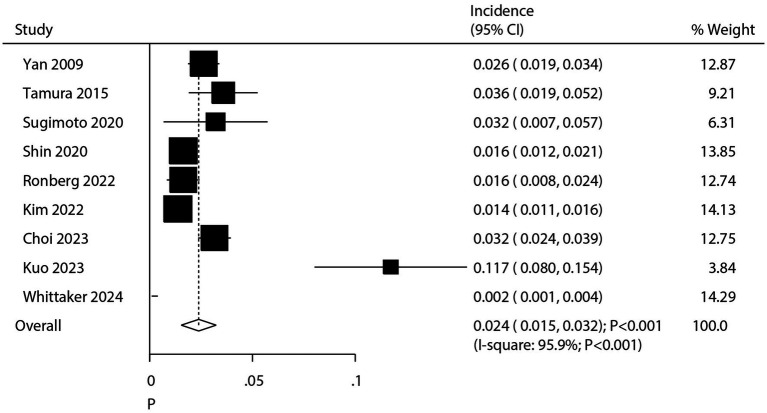
Summary incidence of chronic pulmonary aspergillosis in patients with lung cancer. CI, confidence interval. Incidence was calculated by the number of pulmonary aspergillosis/the number of patients with lung cancer.

After pooling all included studies, we noted the incidence of pulmonary aspergillosis in patients with lung cancer was 2.4% (95% CI: 1.5–3.2%; *p* < 0.001; Figure 2), and significant heterogeneity was observed across included studies (*I*^2^ = 95.9%; *p* < 0.001). Sensitivity analysis revealed that the pooled incidence of pulmonary aspergillosis ranged from 2.0% (95% CI: 1.2–2.8%) to 2.7% (95% CI: 1.6–3.9%) when a single study was sequentially removed (Supplementary Figure S1). Subgroup analysis further suggested higher incidences of pulmonary aspergillosis than corresponding subgroups in the following categories: Asia (2.8%; 95% CI: 2.0–3.7%), diagnosis by serological test (11.7%; 95% CI: 8.0–15.4%), patients with NSCLC and SCLC (3.6%; 95% CI: 2.0–5.2%), patients treated with chemoradiotherapy (5.7%; 95% CI: 1.6–9.7%), and pooled studies of moderate quality (2.9%; 95% CI: 1.7–3.2%) (Table 2).

**Table 2:** Subgroup analyses for the incidence of pulmonary aspergillosis in patients with lung cancer.

Factors	Subgroups	Number of studies	Incidence and 95% CI	*p-*value	*I*^2^ (%)	Q statistic	Interaction test
Country	Asia	7	2.8% (2.0–3.7%)	< 0.001	89.7	< 0.001	< 0.001
	Europe	2	0.9% (0.0–2.2%)	0.200	91.2	0.001	
Diagnostic criteria	Microbiological culture	8	2.0% (1.2–2.8%)	< 0.001	95.6	< 0.001	< 0.001
	Serological test	1	11.7% (8.0–15.4%)	< 0.001	-	-	
Lung cancer	NSCLC	5	1.6% (0.7–2.5%)	< 0.001	95.6	< 0.001	< 0.001
	NSCLC and SCLC	4	3.6% (2.0–5.2%)	< 0.001	90.3	< 0.001	
Treatments	Lung resection	4	1.4% (0.5–2.4%)	0.002	96.5	< 0.001	< 0.001
	Chemoradiotherapy	3	5.7% (1.6–9.7%)	0.006	89.9	< 0.001	
	Not mentioned	2	2.1% (1.2–3.1%)	< 0.001	68.2	0.076	
Study quality	High	4	1.9% (0.8–3.0%)	0.001	97.1	< 0.001	< 0.001
	Moderate	5	2.9% (1.7–3.2%)	< 0.001	88.1	< 0.001	

### 3.4 Predictors for pulmonary aspergillosis in patients with lung cancer

**Figure 3:**
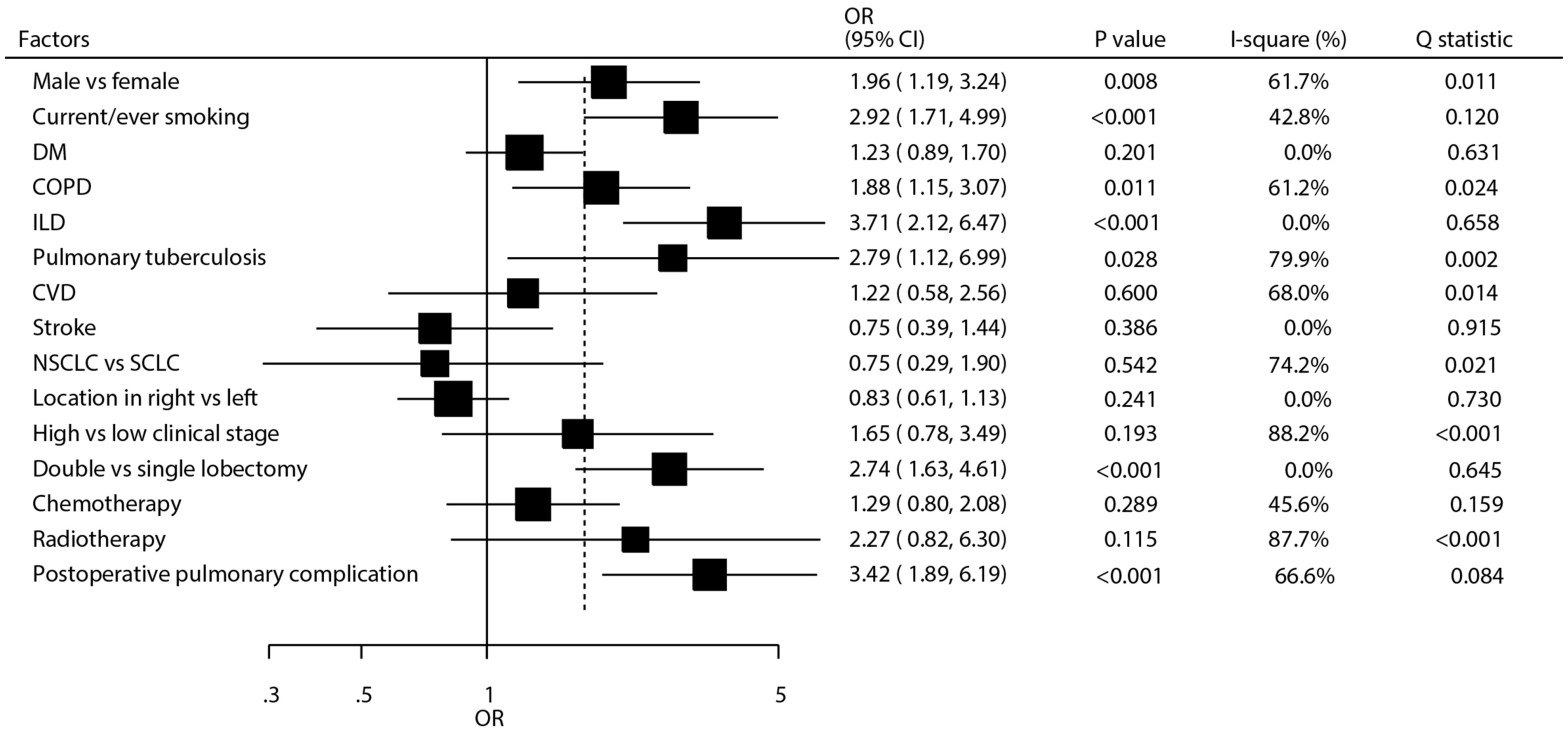
Summary predictors for pulmonary aspergillosis in patients with lung cancer. CI, confidence interval; COPD, chronic obstructive pulmonary disease; CVD, cardiovascular disease; DM, diabetes mellitus; ILD, interstitial lung disease; NSCLC, non-small cell lung cancer; OR, odds ratio; SCLC, small cell lung cancer.

As shown in Figure 3 and Supplementary Figures S2–S18, we investigated the factors influencing the occurrence of pulmonary aspergillosis in lung cancer patients. The summary results indicated that male sex (OR: 1.96; 95% CI: 1.19–3.24; *p* = 0.008), current or past smoking (OR: 2.92; 95% CI: 1.71–4.99; *p* < 0.001), chronic obstructive pulmonary disease (COPD) (OR: 1.88; 95% CI: 1.15–3.07; *p* = 0.011), interstitial lung disease (ILD) (OR: 3.71; 95% CI: 2.12–6.47; *p* < 0.001), pulmonary tuberculosis (OR: 2.79; 95% CI: 1.12–6.99; *p* = 0.028), treatment with double lobectomy (OR: 2.74; 95% CI: 1.63–4.61; *p* < 0.001), and postoperative pulmonary complications (OR: 3.42; 95% CI: 1.89–6.19; *p* < 0.001) were all associated with an increased risk of pulmonary aspergillosis. However, diabetes mellitus (DM), cardiovascular disease (CVD) history, stroke, cancer type, cancer location, clinical stage, and whether patients received chemotherapy or radiotherapy were not associated with the risk of pulmonary aspergillosis. There were significant heterogeneity for male sex (*I*^2^ = 61.7%; *p* = 0.011), COPD (*I*^2^ = 61.2%; *p* = 0.024), pulmonary tuberculosis (*I*^2^ = 79.9%; *p* = 0.002), CVD (*I*^2^ = 68.0%; *p* = 0.014), cancer type (*I*^2^ = 74.2%; *p* = 0.021), clinical stage (*I*^2^ = 88.2%; *p* < 0.001), radiotherapy (*I*^2^ = 87.7%; *p* < 0.001), and postoperative pulmonary complications (*I*^2^ = 66.6%; *p* = 0.084). The sensitivity analysis revealed that the pooled conclusion regarding the association of COPD with the risk of pulmonary aspergillosis in patients with lung cancer was variable. In contrast, the pooled conclusions regarding other predictors were stable.

### 3.5 Publication bias

**Figure 4:**
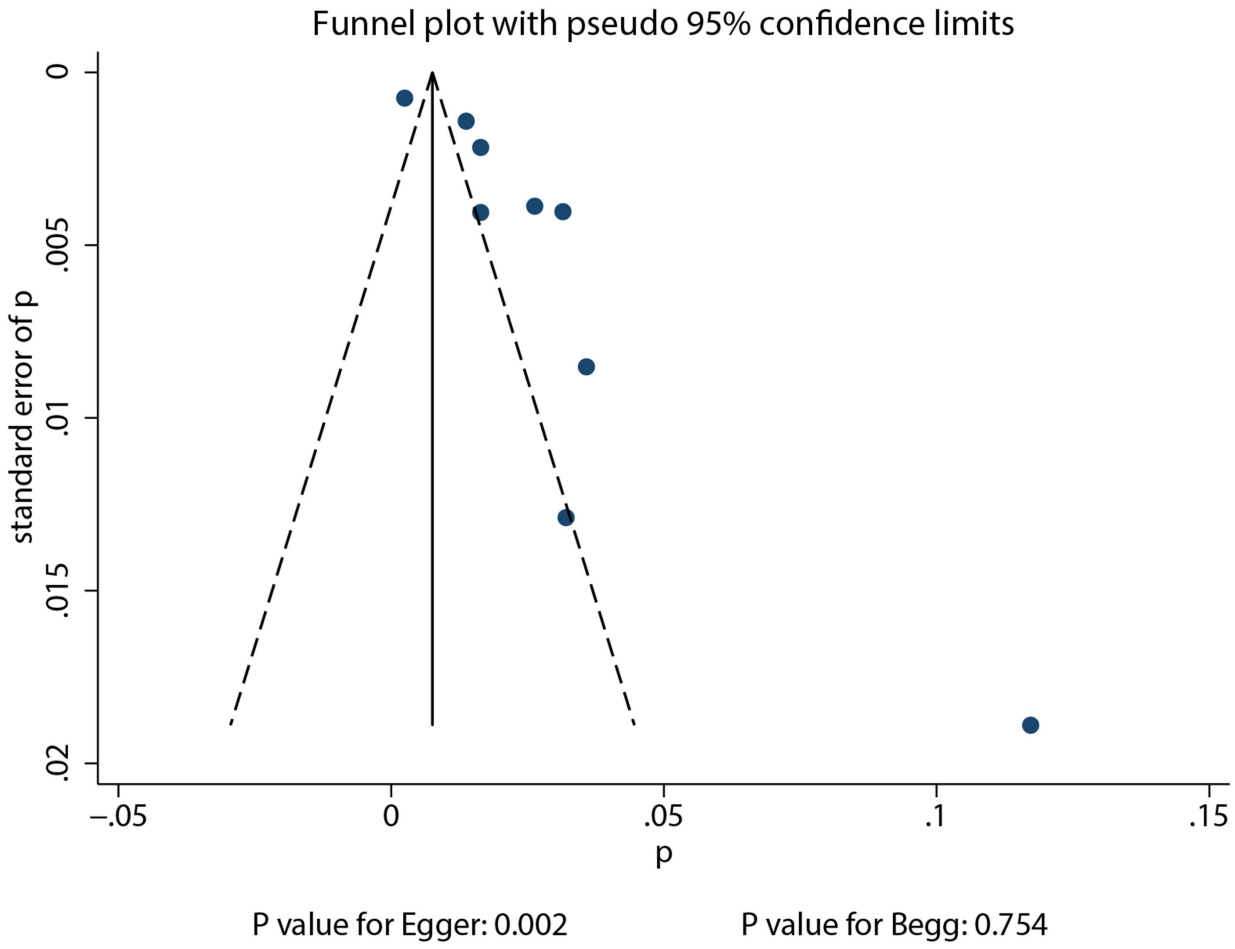
Funnel plot for pulmonary aspergillosis incidence.

Reviewing the funnel plots did not rule out potential publication bias for the incidence of pulmonary aspergillosis in patients with lung cancer (Figure 4). Begg’s test (*p* = 0.754) indicated no significant publication bias, while Egger’s test (*p* = 0.002) suggested potential publication bias for pulmonary aspergillosis in patients with lung cancer. After adjusting for potential confounding factors, the conclusion indicated stability using the trim and fill method (30). There was no significant publication bias for gender (Egger’s *p* = 0.637; Begg’s *p* = 0.174), current or past smoking (Egger’s *p* = 0.921; Begg’s *p* = 1.000), DM (Egger’s *p* = 0.236; Begg’s *p* = 0.707), and COPD (Egger’s *p* = 0.800; Begg’s *p* = 1.000) with respect to the risk of pulmonary aspergillosis. However, potential publication bias was observed for the association between clinical stage and the risk of pulmonary aspergillosis (Egger’s *p* = 0.041; Begg’s *p* = 0.133). The conclusion was not altered after adjusting for publication bias using the trim and fill method (30).

## 4 Discussion

This study was the first to employ a meta-analysis approach to investigate the incidence of pulmonary aspergillosis in lung cancer patients and its associated risk factors. Through a systematic review, we included a total of 9 studies, encompassing 20,138 lung cancer patients, and 337 patients diagnosed with pulmonary aspergillosis. Although we did not restrict the study design during the initial search, only retrospective studies met our inclusion criteria. The rarity of pulmonary aspergillosis in lung cancer patients has made it difficult to conduct prospective studies, which typically require substantial resources, extended follow-up periods, and large sample sizes to yield meaningful results. Notably, there was significant variability in the severity of illness and baseline characteristics among the patients included in these studies. We noted the incidence of pulmonary aspergillosis in lung cancer patients was 2.4% (95% CI: 1.5–3.2%). Moreover, the risk factors for pulmonary aspergillosis in patients with lung cancer included male sex, current or past smoking, COPD, ILD, pulmonary tuberculosis, double lobectomy, and postoperative pulmonary complications.

Our research revealed an incidence rate of 2.4% for pulmonary aspergillosis among lung cancer patients, with rates in the included studies ranging from 0.2 to 11.7%. The majority of studies reported incidence rates between 1 and 3%. A primary reason contributing to the higher reported incidence, particularly in one study, was the administration of a galactomannan test on 290 high-risk patients out of 2,543 with advanced lung cancer, leading to a relatively higher reported incidence of pulmonary aspergillosis (28). The subgroup analysis revealed that the incidence of pulmonary aspergillosis was higher in Asia, among patients diagnosed with pulmonary aspergillosis using serological tests, in those with both NSCLC and SCLC, in patients treated with chemoradiotherapy, and in pooled studies of moderate quality than corresponding subgroups. The potential reasons for this include variations in diagnostic practices and accessibility to medical resources across regions, which can impact disease identification and reporting rates. In addition, Asian patients may be more prone to developing pulmonary aspergillosis due to immune suppression resulting from specific treatment regimens (31). Environmental factors play a crucial role in the regional differences in *Aspergillus* species distribution. In Asia, the climate is often more humid and warmer in many areas than in Europe. Differences in air pollution levels can impact fungal survival and distribution. Polluted air can damage the respiratory mucosa of lung cancer patients, making them more susceptible to *Aspergillus* infection. The pollutants may also interact with the fungal spores, affecting their viability and the likelihood of causing infection. Demographic factors can also contribute to regional differences. Population density varies significantly between regions. In highly populated Asian cities, the proximity of individuals can lead to increased exposure to *Aspergillus* spores. Moreover, differences in lifestyle and occupation can influence exposure. In some Asian countries, certain occupations such as farming or working in traditional markets may expose individuals to a higher concentration of *Aspergillus* spores. Genetic factors also play a role, and these genetic differences could make Asian lung cancer patients more or less susceptible to certain *Aspergillus* species compared to European patients. Serological tests are more likely to detect early-stage or subclinical infections, as they can detect the presence of fungal antigens in the blood. This allows for the detection of cases before the development of obvious clinical symptoms or before the fungus can be cultured. As a result, serological tests contribute to higher incidence estimates compared to diagnostic methods that rely on more advanced disease manifestations. SCLC is often more aggressive, and patients with SCLC may receive more intensive chemotherapy and radiotherapy regimens. These treatments can cause profound immunosuppression, including a decrease in the number and function of lymphocytes. The combined effect of the disease-related immune suppression and the intensive treatment-induced immunosuppression in patients with both NSCLC and SCLC makes them more prone to pulmonary aspergillosis (32). Finally, chemoradiotherapy can induce oxidative stress in the body. High levels of reactive oxygen species generated during treatment can damage cells and tissues, including immune cells. Increased oxidative stress can also enhance the inflammatory response in the lungs. This chronic inflammation can create a microenvironment that is more conducive to *Aspergillus* growth and infection (33).

Our study found that risk factors for pulmonary aspergillosis in lung cancer patients included male sex, current or past smoking, COPD, ILD, pulmonary tuberculosis, double lobectomy, and postoperative pulmonary complications. Several potential reasons could explain these results: (1) Males and females exhibit physiological differences in their immune responses, with males potentially having reduced resistance to *Aspergillus* infections due to hormonal levels, genetic backgrounds, or other physiological traits (34); (2) long-term smoking suppresses the immune system, particularly weakening the body’s defense mechanisms against pathogens, including reducing the functionality of macrophages and lymphocytes, thereby decreasing resistance to *Aspergillus* infections. Furthermore, smoking can lead to imbalances in pulmonary cytokines (proteins involved in immune responses), which may disrupt normal immune responses and create conditions conducive to the development of aspergillosis (35); (3) COPD-induced airway inflammation, mucus hypersecretion, structural alterations, and lung damage create a susceptible environment for *Aspergillus*, facilitating local infections (36); (4) ILD leads to inflammation and fibrosis of the lung interstitium, impairing gas exchange and also disrupting the typical structure of lung tissue, thereby providing a conducive environment for colonization by microorganisms (37); (5) inflammation and tissue damage caused by tuberculosis can lead to the formation of scars and cavities within the lung parenchyma. These structural alterations create physical spaces that facilitate colonization and growth of microorganisms (38); (6) double lobectomy significantly reduces the total lung tissue volume, leading to severe impairment of lung function, including diminished capacity for gas exchange and a decline in the respiratory tract’s clearance mechanisms. This renders the remaining lung tissue more vulnerable to infection by pathogens (22), and (7) lung cancer surgeries often entail the resection of lung tissue, which may inflict damage to the surrounding healthy lung tissue, leading to a decline in lung function. Postoperative atelectasis, reduced lung compliance, and impaired gas exchange and respiratory mechanics collectively contribute to a condition that facilitates colonization by microorganisms.

Considering the identified risk factors for pulmonary aspergillosis in lung cancer patients, routine screening using biomarkers such as galactomannan or *β*-D-glucan could be considered in high-risk populations. Patients with risk factors such as male sex, current or past smoking, COPD, interstitial lung disease, pulmonary tuberculosis, double lobectomy, or postoperative pulmonary complications are at increased risk. Galactomannan, a cell-wall component of *Aspergillus*, can be detected in serum or other body fluids. Its measurement has shown potential in the early detection of invasive aspergillosis. Similarly, β-D-glucan, a polysaccharide present in the cell walls of many fungi, including *Aspergillus*, can also be used as a screening biomarker. Routine screening with these biomarkers in high-risk lung cancer patients could lead to earlier diagnosis and potentially more effective treatment. However, it is important to note that both tests have limitations, such as false-positive results, especially in patients with other conditions that can cause cross-reactivity. Further research is needed to determine the optimal screening intervals and cutoff values for these biomarkers in the context of lung cancer patients at high risk of pulmonary aspergillosis.

Since the included studies were retrospective and the majority of them did not comprehensively adjust for potential confounders, unmeasured confounding is a significant concern. Unmeasured factors, such as lifestyle factors (e.g., diet and exercise), environmental exposures (e.g., exposure to other fungi or pollutants in the living or working environment), and genetic factors, may influence both the development of lung cancer and the susceptibility to pulmonary aspergillosis. These unmeasured confounders could potentially distort our observed associations between risk factors and the development of pulmonary aspergillosis.

Several shortcomings of this study should be acknowledged. First, this study’s analysis is based on a retrospective cohort study, which inherently limits our ability to establish causation and is inevitably subject to selection and recall biases. Second, the lung cancer patients included in the study had varying histological types and disease severity and received different treatments, all of which could influence the risk of developing pulmonary aspergillosis. Third, the investigation of risk factors was based on crude data, and adjusted effect estimates were not obtainable. Fourth, the use of limited databases for the search represents a significant limitation of this study, potentially obscuring the actual disease burden and introducing publication bias. Fifth, variations in the diagnostic approaches for pulmonary aspergillosis across different studies may impact the accuracy of reported incidence rates among lung cancer patients. Sixth, the incidence and risk factors for pulmonary aspergillosis in lung cancer patients may be influenced by lung cancer type, but the incidence rates and associated risk factors for pulmonary aspergillosis by lung cancer type were not available. Seventh, the potential confounding effects of differences in lung cancer stage and histological subtypes were not fully addressed in the exploratory analysis. Eighth, although identifying the specific *Aspergillus* spp. causing pulmonary aspergillosis in lung cancer patients is important, the included studies did not provide this information. Future research should focus on using more advanced diagnostic techniques, such as molecular identification methods, to determine the species of *Aspergillus* involved. This could potentially provide more insights into the pathogenesis and treatment of pulmonary aspergillosis in this patient population. Finally, the analysis of this study, based on published articles, was limited by the unpublished data and restricted detailed analyses.

## 5 Conclusion

This systematic review and meta-analysis represents one of the first comprehensive attempts to explore the incidence and predictors of pulmonary aspergillosis in lung cancer patients. Despite the limitations associated with the retrospective nature of the included studies, our findings provide valuable insights into the field. The overall incidence of pulmonary aspergillosis in lung cancer patients, estimated at 2.4%, highlights that this is a non-negligible complication. The identification of male sex, current or past smoking, COPD, ILD, pulmonary tuberculosis, double lobectomy, and postoperative pulmonary complications as risk factors for pulmonary aspergillosis in lung cancer patients provides a basis for targeted screening and preventive measures. These factors, either directly or indirectly, compromise the immune system or the integrity of the lung tissue, creating a conducive environment for *Aspergillus* infection. However, our study has limitations, primarily due to the retrospective nature of the included studies, potential unmeasured confounding, and variations in diagnostic criteria. Future prospective studies using standardized diagnostic methods and carefully adjusting for confounders are urgently needed to validate and expand upon our findings. These studies should also explore the underlying biological mechanisms linking these risk factors to the development of pulmonary aspergillosis, potentially leading to more effective preventive and treatment strategies.

## Data Availability

The original contributions presented in the study are included in the article/Supplementary material, further inquiries can be directed to the corresponding authors.
