# Supplementary material for: Incidence and predictors of pulmonary aspergillosis in patients with lung cancer: a systematic review and meta-analysis
Source: Front Med (Lausanne). 2025 Apr 28;12:1560288. doi: 10.3389/fmed.2025.1560288 (PMC12066252; doi:10.3389/fmed.2025.1560288)
Supplement: Supplementary file 1 [file Table_1.DOCX]

**STATA commands**

For incidence of CPA in patients with lung cancer:

gen p=case/n

gen se=sqrt(p*(1-p)/n)

metan p se,random effect(P) xlabel(0, 0.2, 0.4) label (namevar=study)

For predictors of CAP in patients with lung cancer:

gen logor=log(or)

gen logorl=log(orl)

gen logoru=log(oru)

metan7 logor logorl logoru, eform random xlab (0.3, 0.5, 1.0, 2.0) effect (OR) label (namevar=study)

Table S1. Quality scores of observational studies using Newcastle-Ottawa Scale.

| Study | Selection | | | | Comparability | Outcome | | | NOS |
| --- | --- | --- | --- | --- | --- | --- | --- | --- | --- |
|  | Representativeness of the exposed cohort | Selection of the non exposed cohort | Ascertainment  of exposed | Demonstration that outcomes was not present at start of study | Comparability on the basis of the design or analysis | Assessment of outcome | Adequate follow-up duration | Adequate follow-up rate | Overall score |
| Yan 2009 [21] | * | * |  | * | * | * | * |  | ****** |
| Tamura 2015 [22] | * | * |  | * | * | * | * | * | ******* |
| Sugimoto 2020 [23] |  | * |  | * | * | * | * |  | ***** |
| Shin 2020 [24] | * |  |  | * | * | * | * | * | ****** |
| Rønberg 2022 [25] |  | * |  | * | * | * | * |  | ***** |
| Kim 2022 [26] | * | * |  | * | * | * | * | * | ******* |
| Choi 2023 [27] | * | * |  | * | * | * | * | * | ******* |
| Kuo 2023 [28] |  | * |  | * | * | * | * | * | ****** |
| Whittaker 2024 [29] | * |  | * | * | * | * | * | * | ******* |


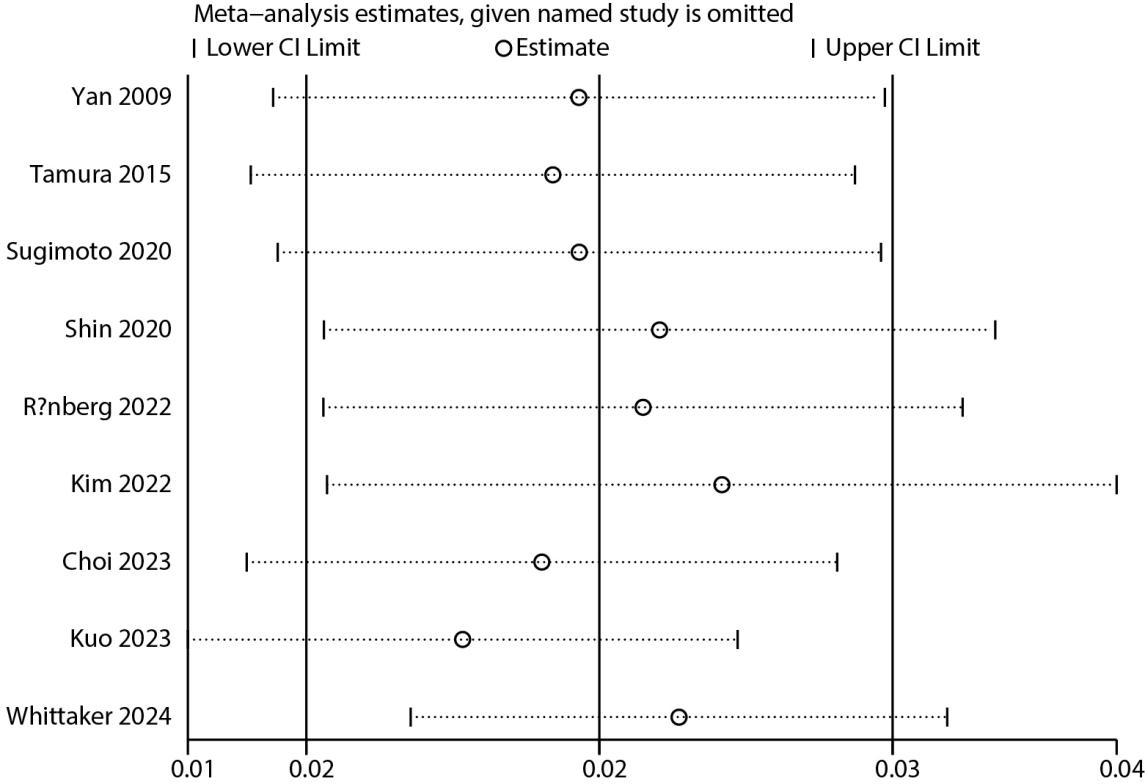


Figure S1. Sensitivity analysis for the incidence of CPD in patients with lung cancer


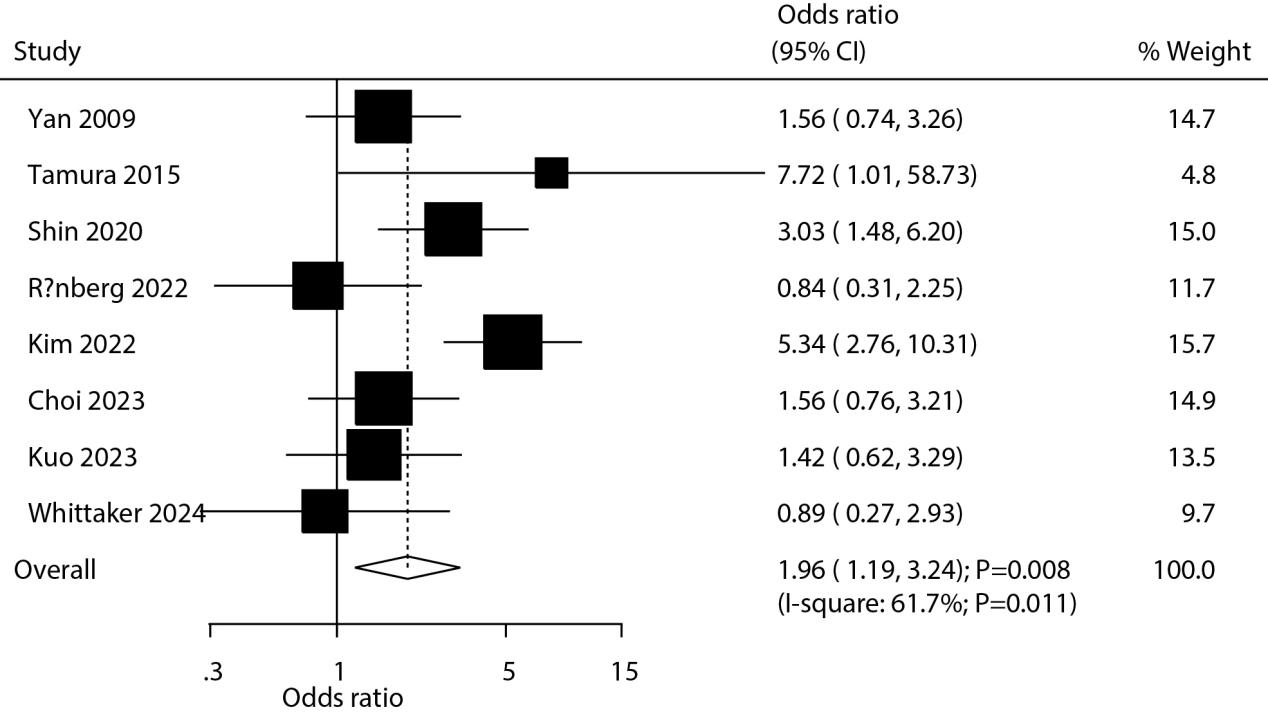


Figure S2. Male vs female on the risk of CPD in patients with lung cancer


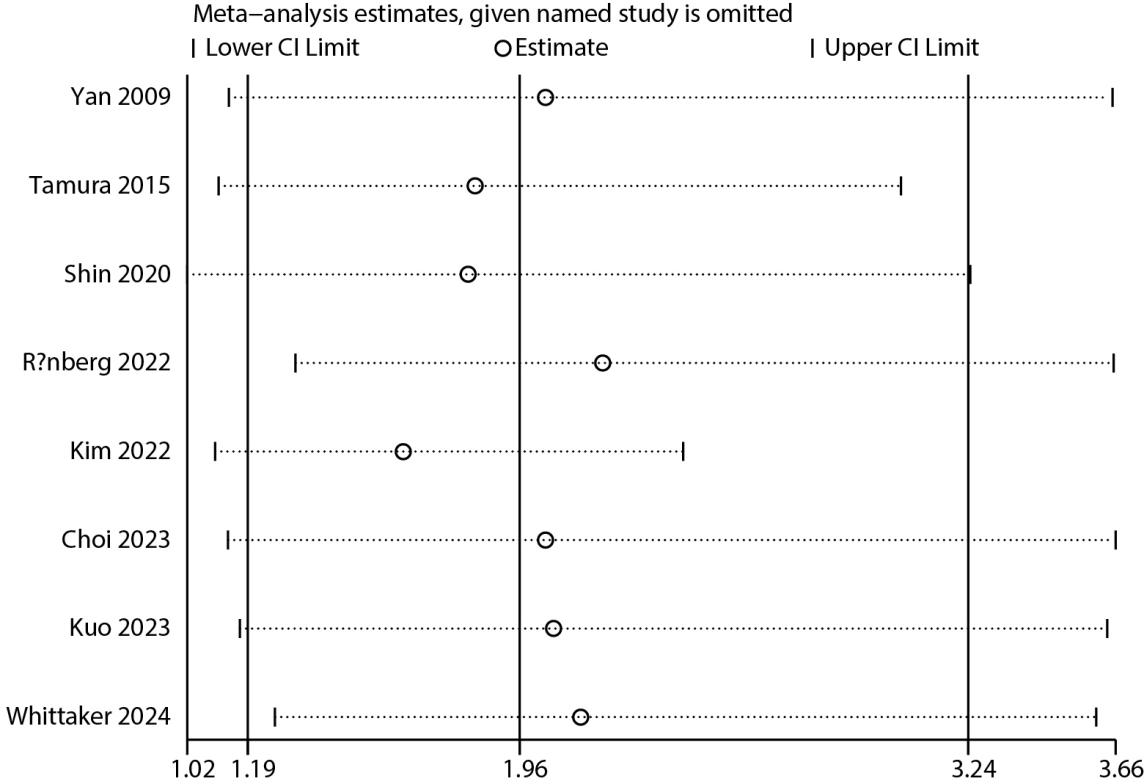


Figure S3. Sensitivity analysis for male vs female on the risk of CPD in patients with lung cancer


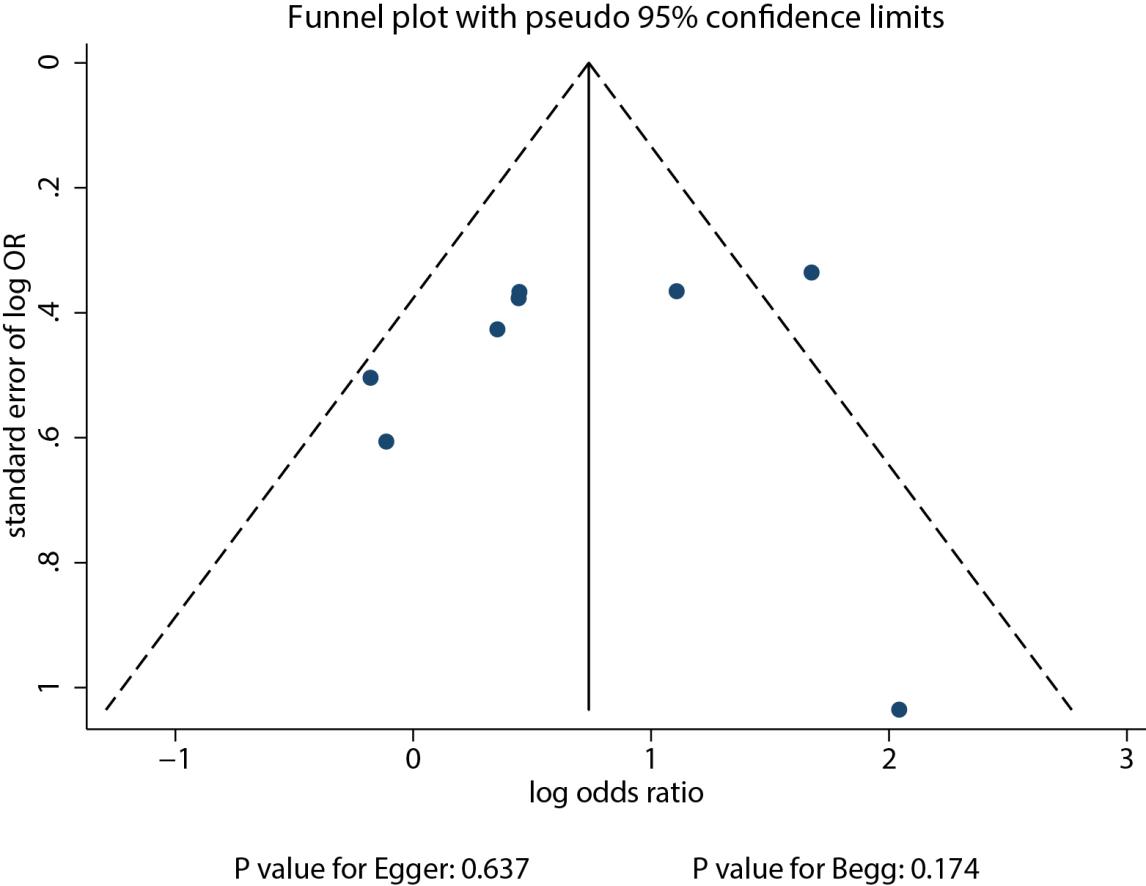


Figure S4. Funnel plot for male vs female on the risk of CPD in patients with lung cancer


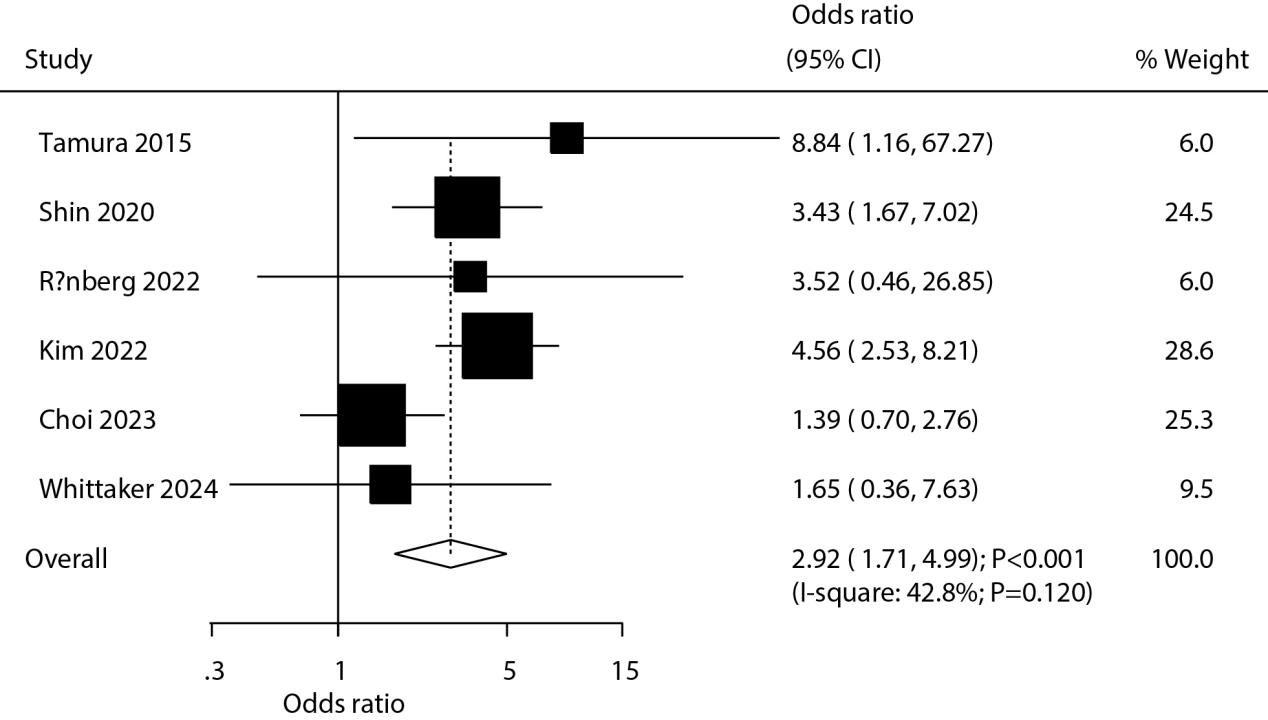


Figure S5. Association of current/ever smoking with the risk of CPD in patients with lung cancer


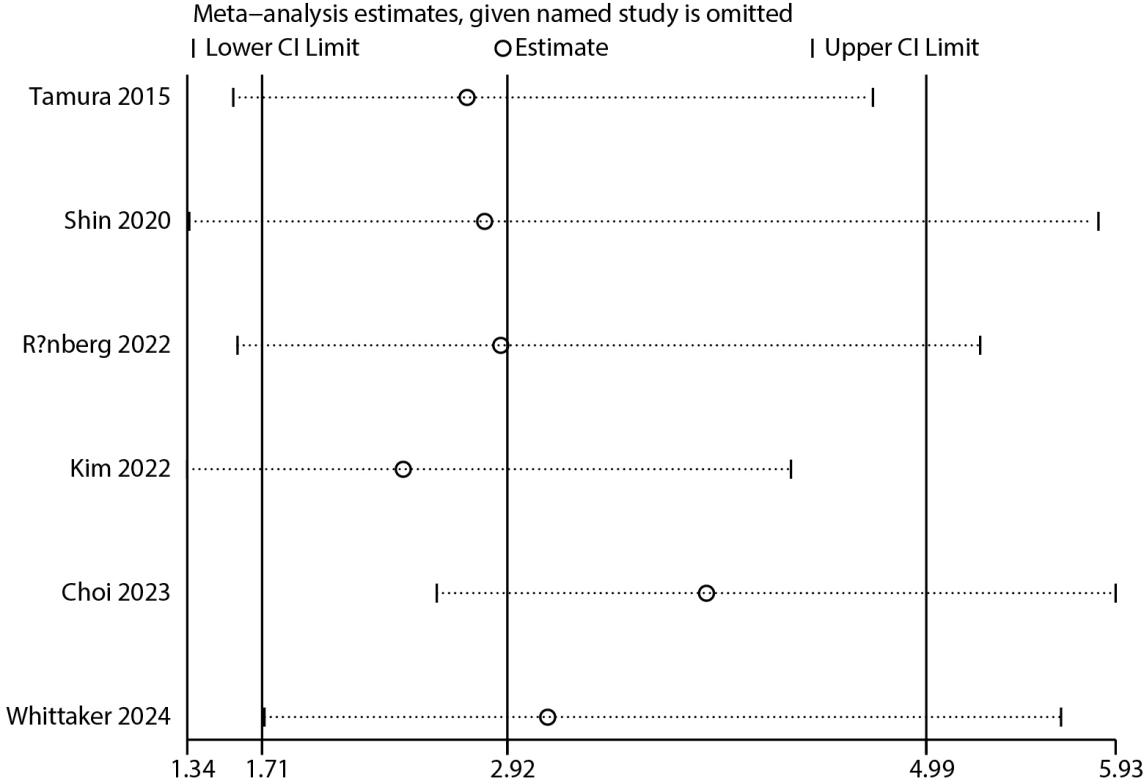


Figure S6. Sensitivity analysis for the association of current/ever smoking with the risk of CPD in patients with lung cancer


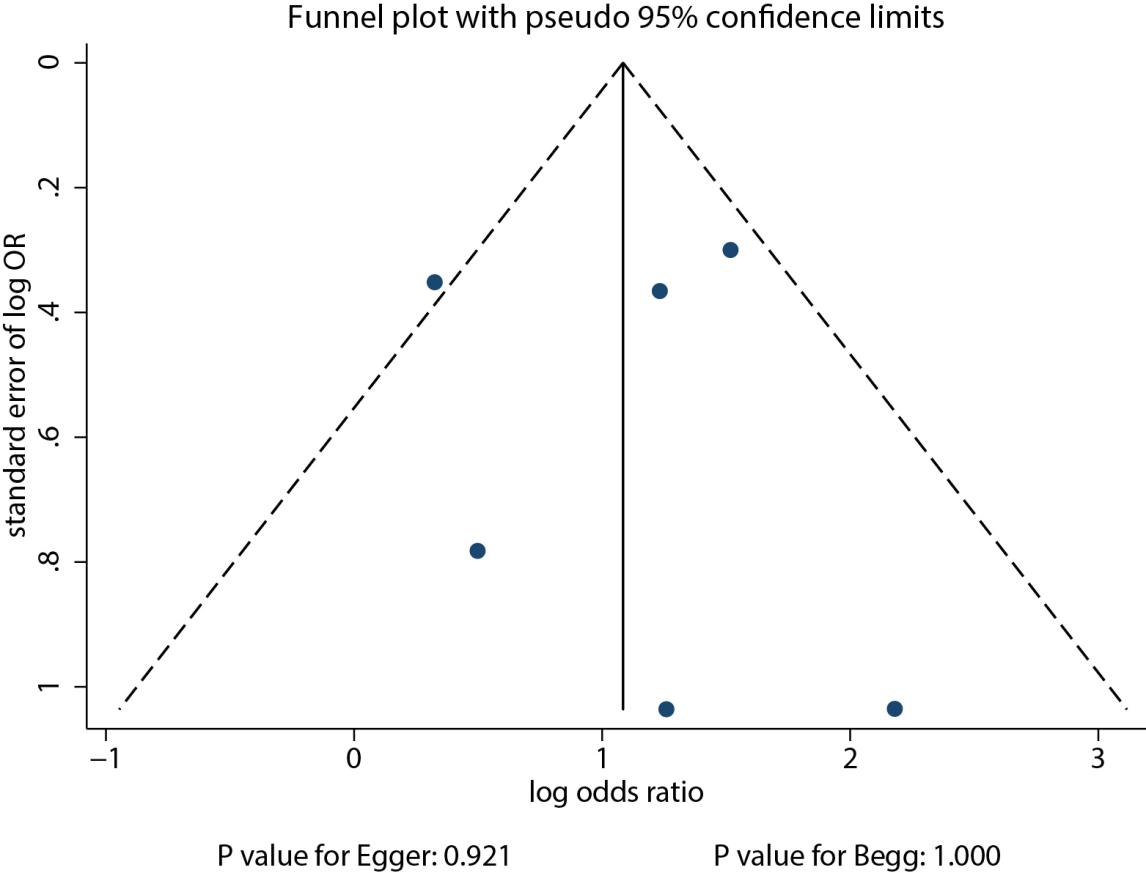


Figure S7. Funnel plot for the association of current/ever smoking with the risk of CPD in patients with lung cancer


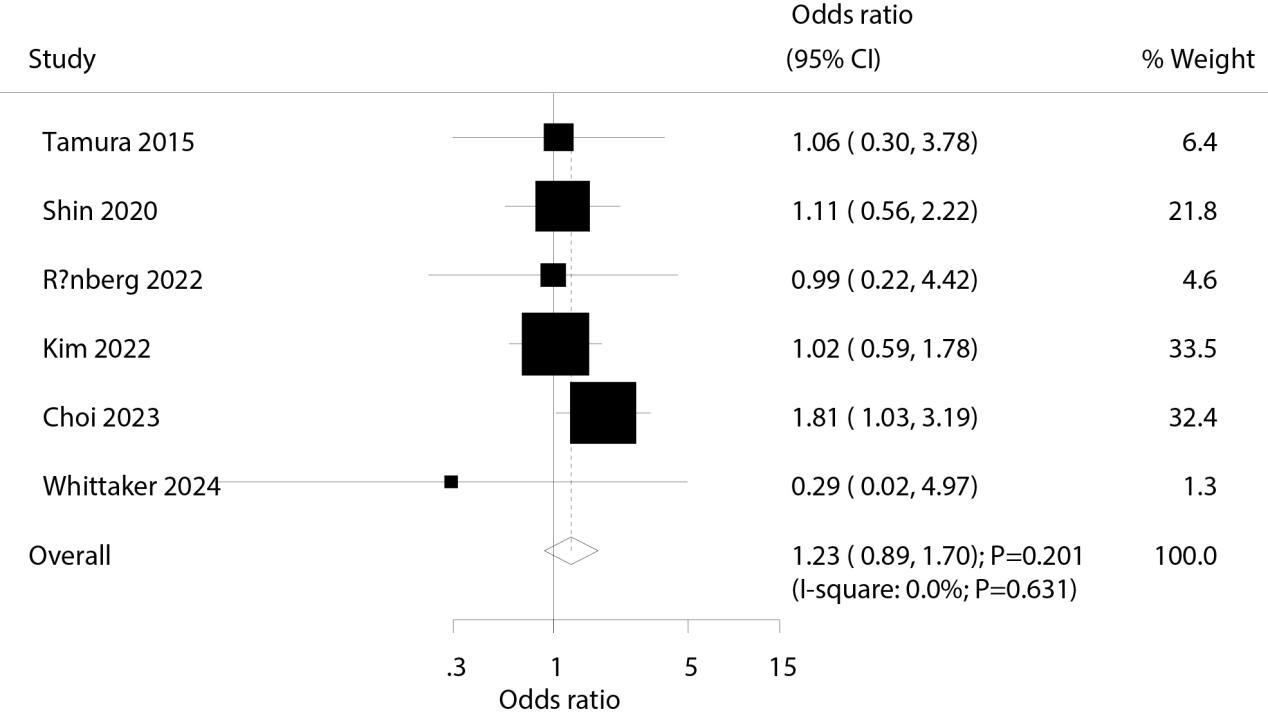


Figure S8. Association of DM with the risk of CPD in patients with lung cancer


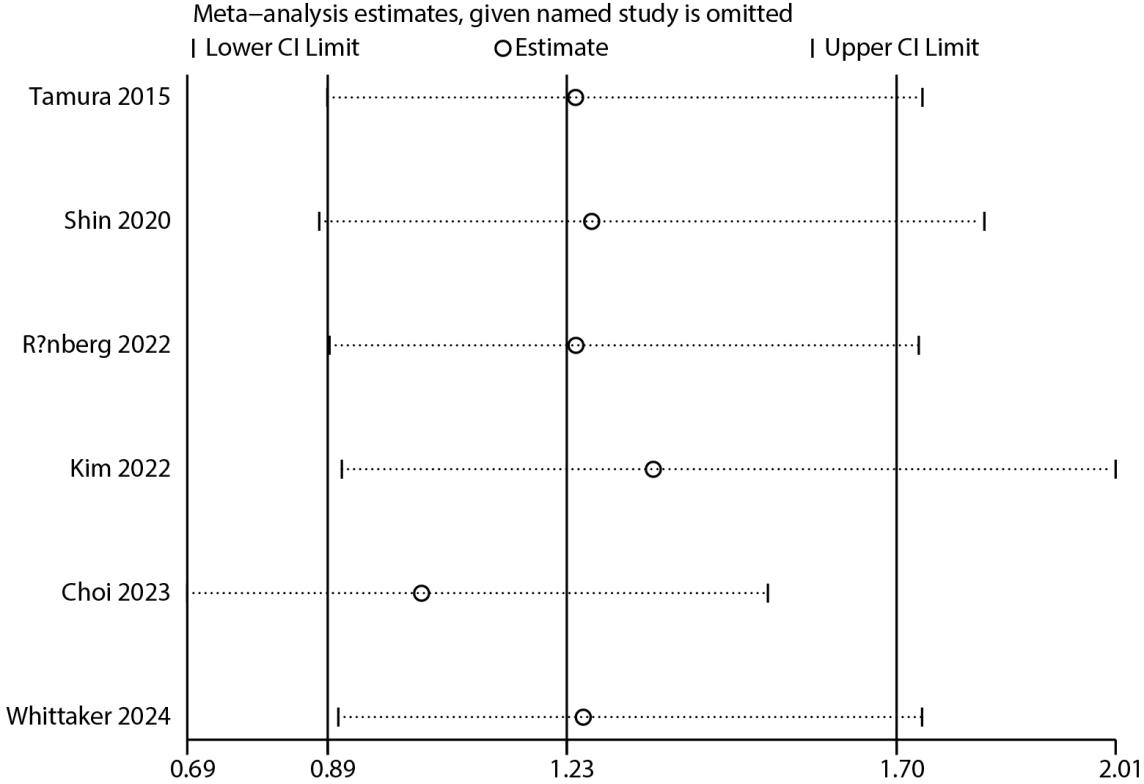


Figure S9. Sensitivity analysis for the association of DM with the risk of CPD in patients with lung cancer


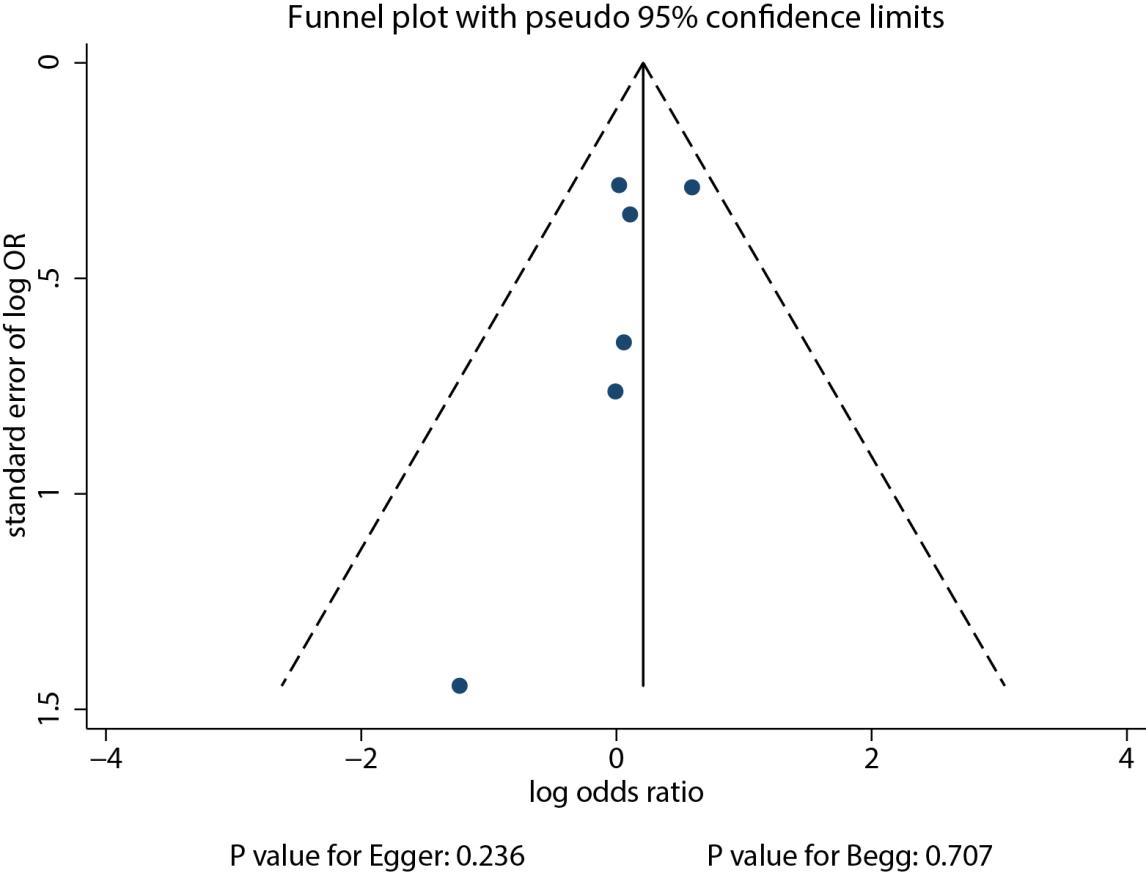


Figure S10. Funnel plot for the association of DM with the risk of CPD in patients with lung cancer


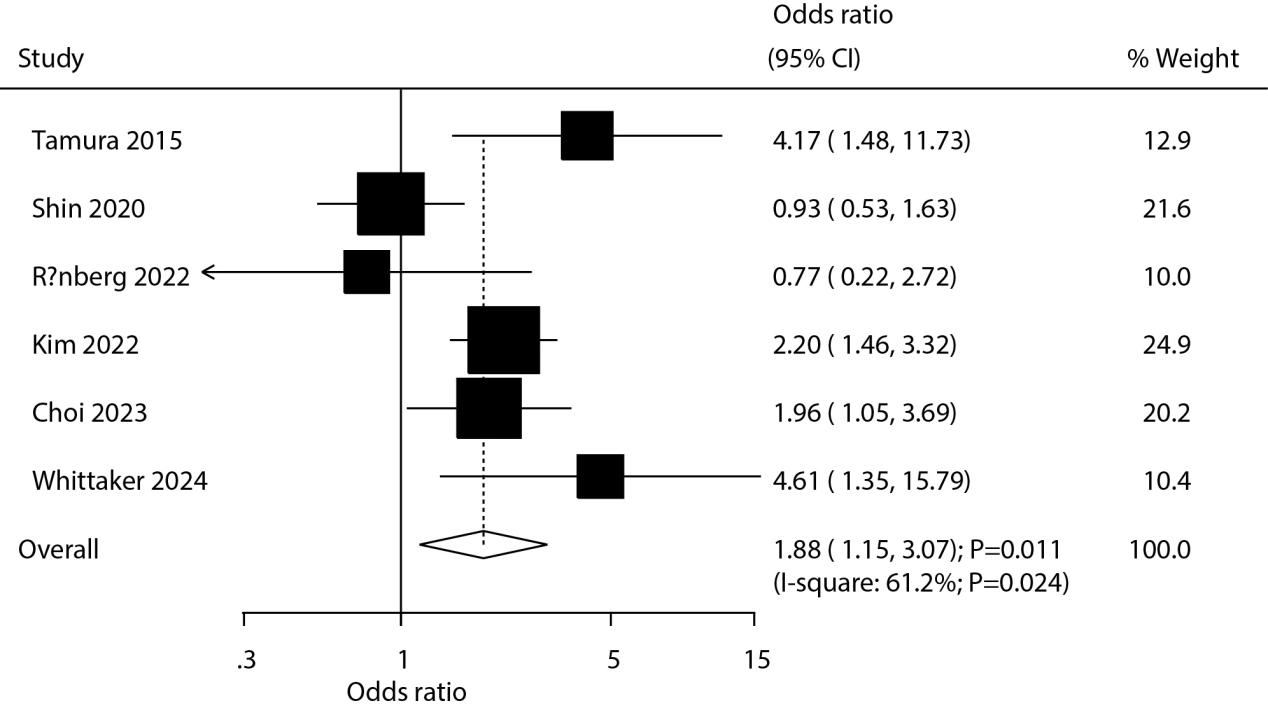


Figure S11. Association of COPD with the risk of CPD in patients with lung cancer


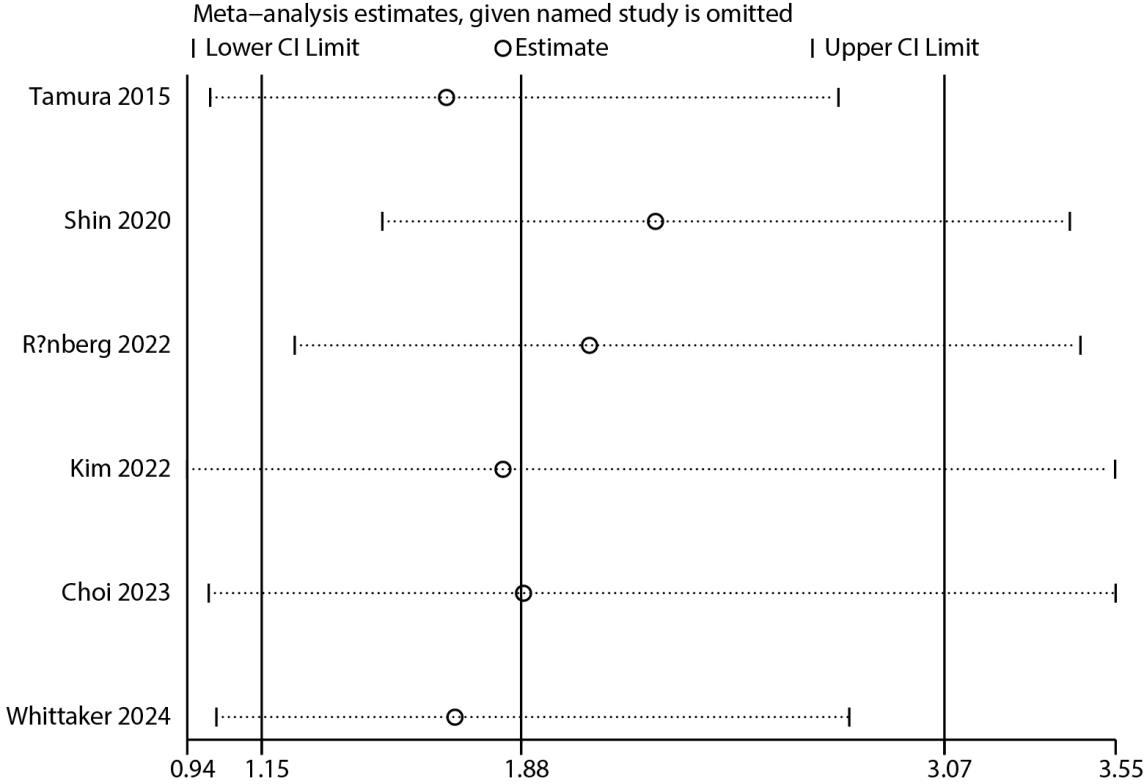


Figure S12. Sensitivity analysis for the association of COPD with the risk of CPD in patients with lung cancer


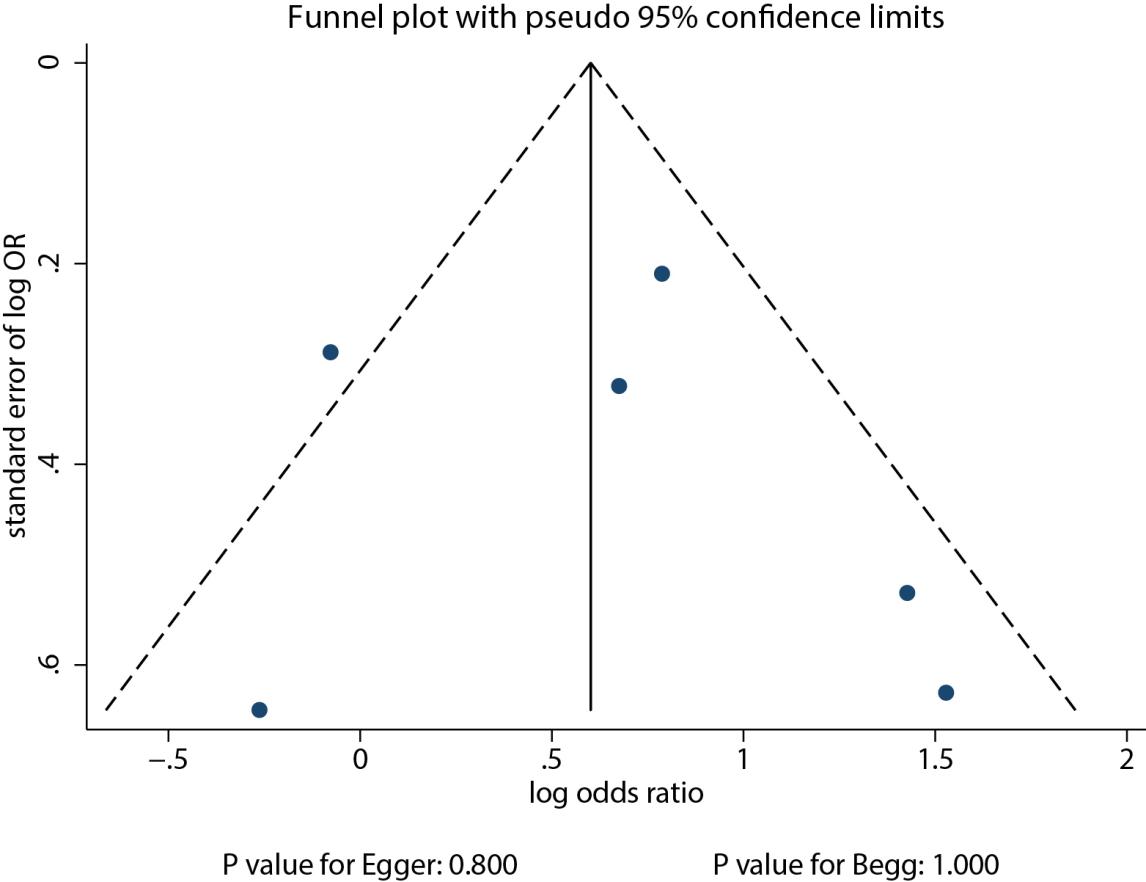


Figure S13. Funnel plot for the association of COPD with the risk of CPD in patients with lung cancer


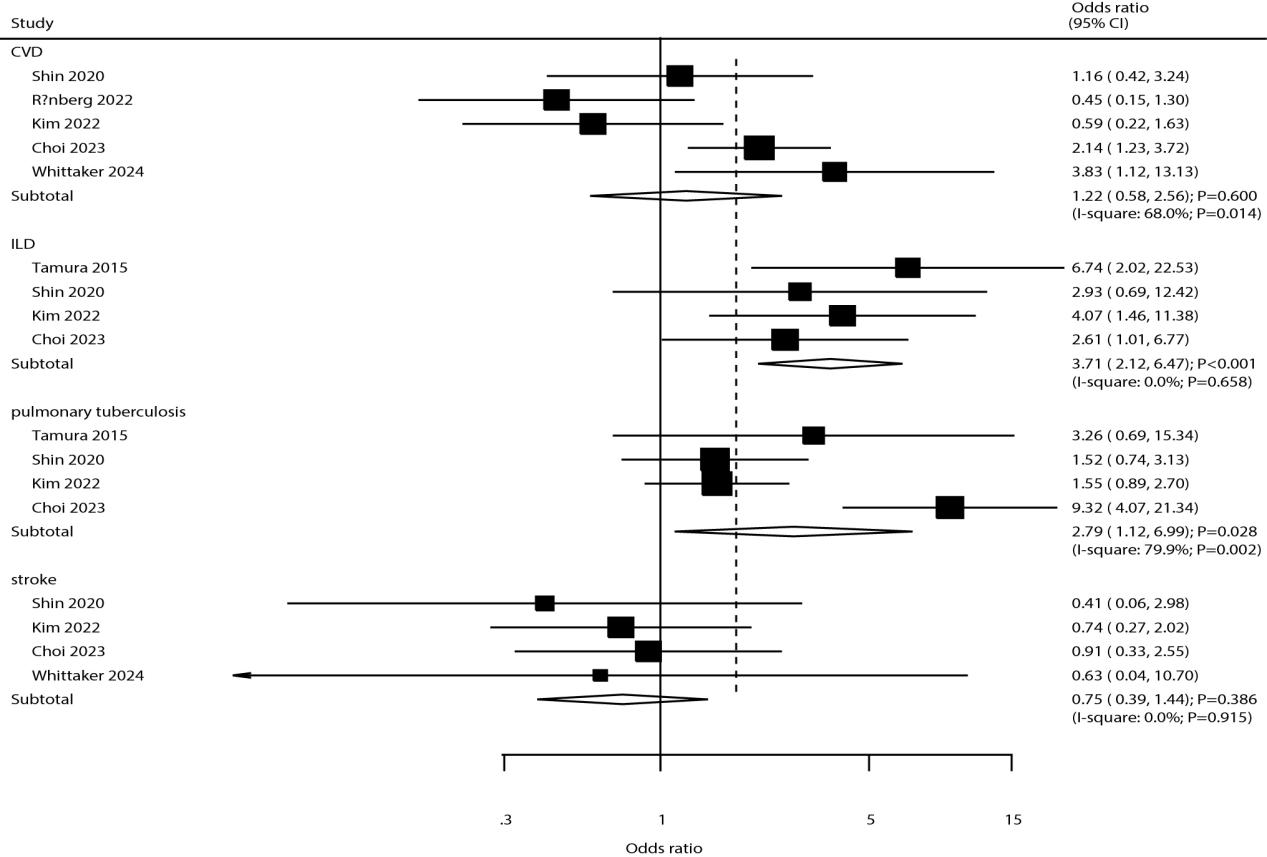


Figure S14. Association of CVD, ILD, pulmonary tuberculosis, and stroke with the risk of CPD in patients with lung cancer


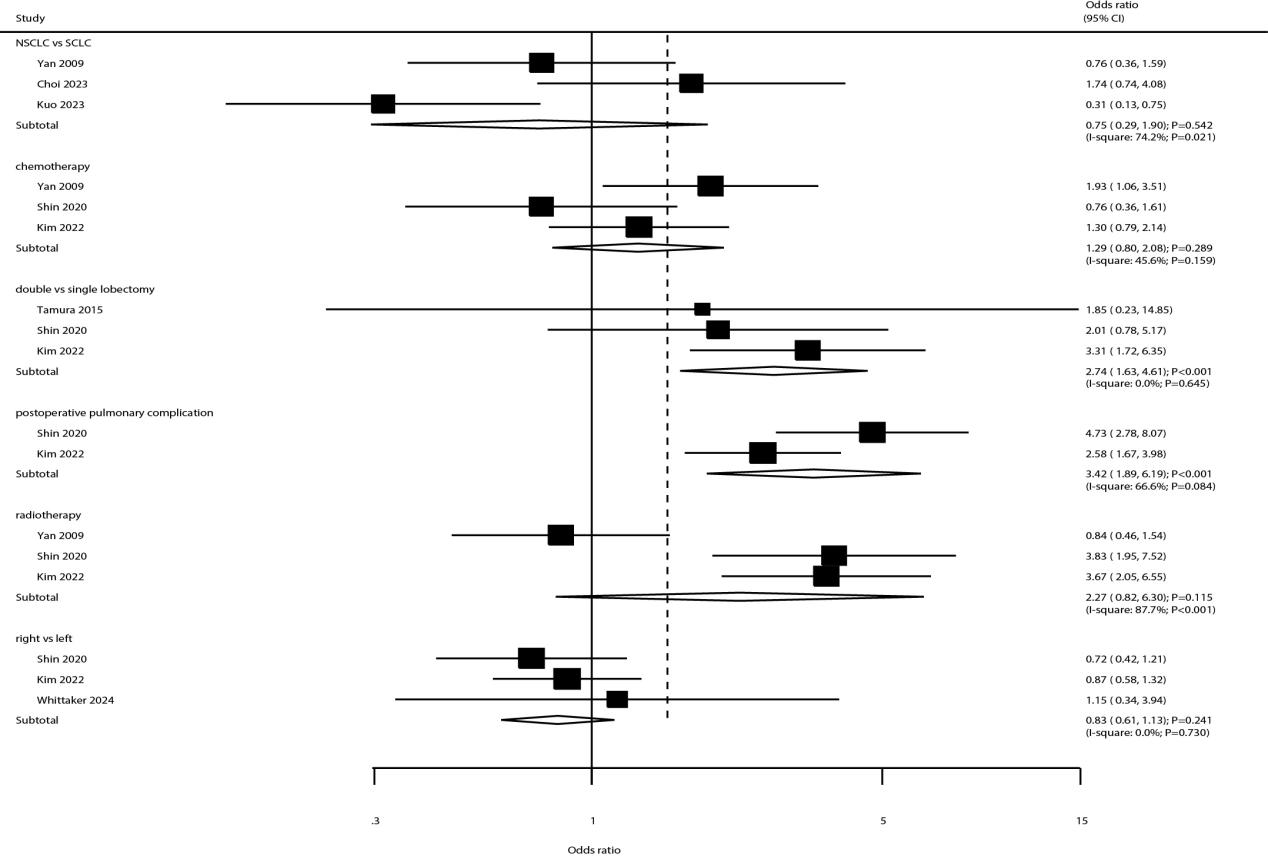


Figure S15. Association of type of lung cancer, chemotherapy, lobectomy, postoperative pulmonary complication, radiotherapy, and cancer location with the risk of CPD in patients with lung cancer


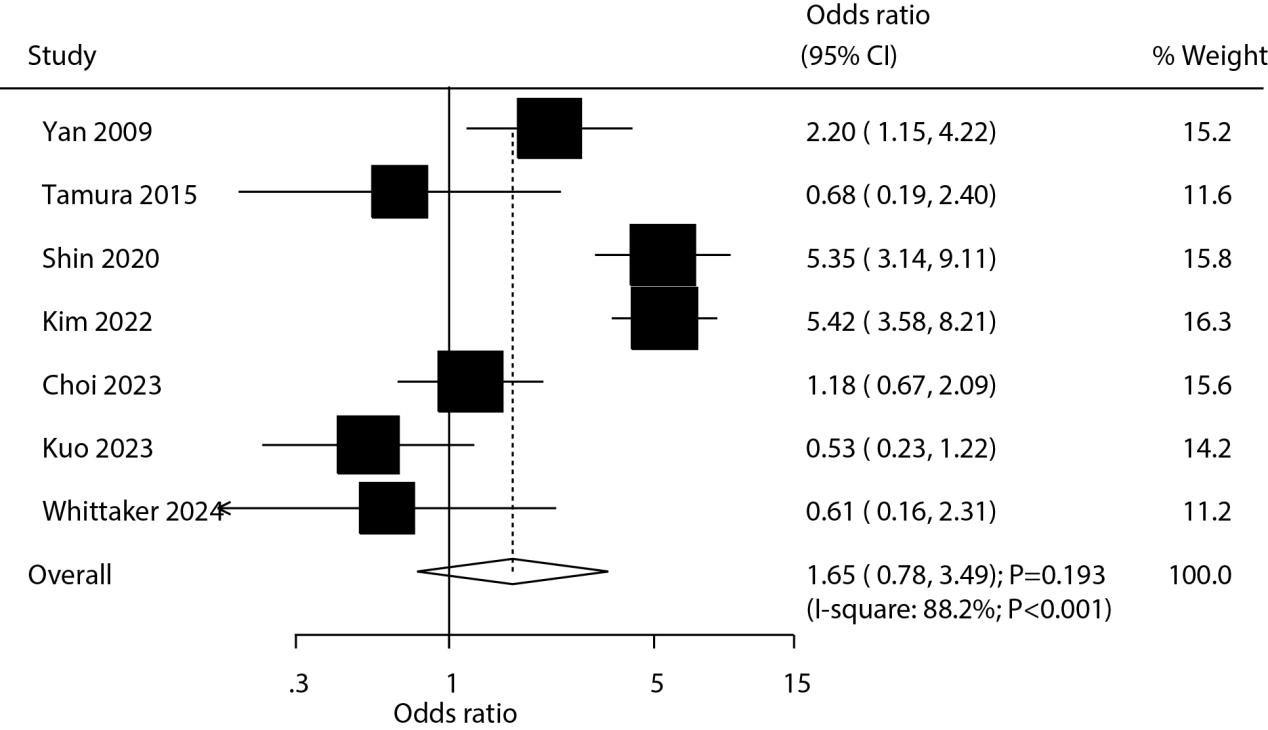


Figure S16. Association of clinical stage of lung cancer with the risk of CPD in patients with lung cancer


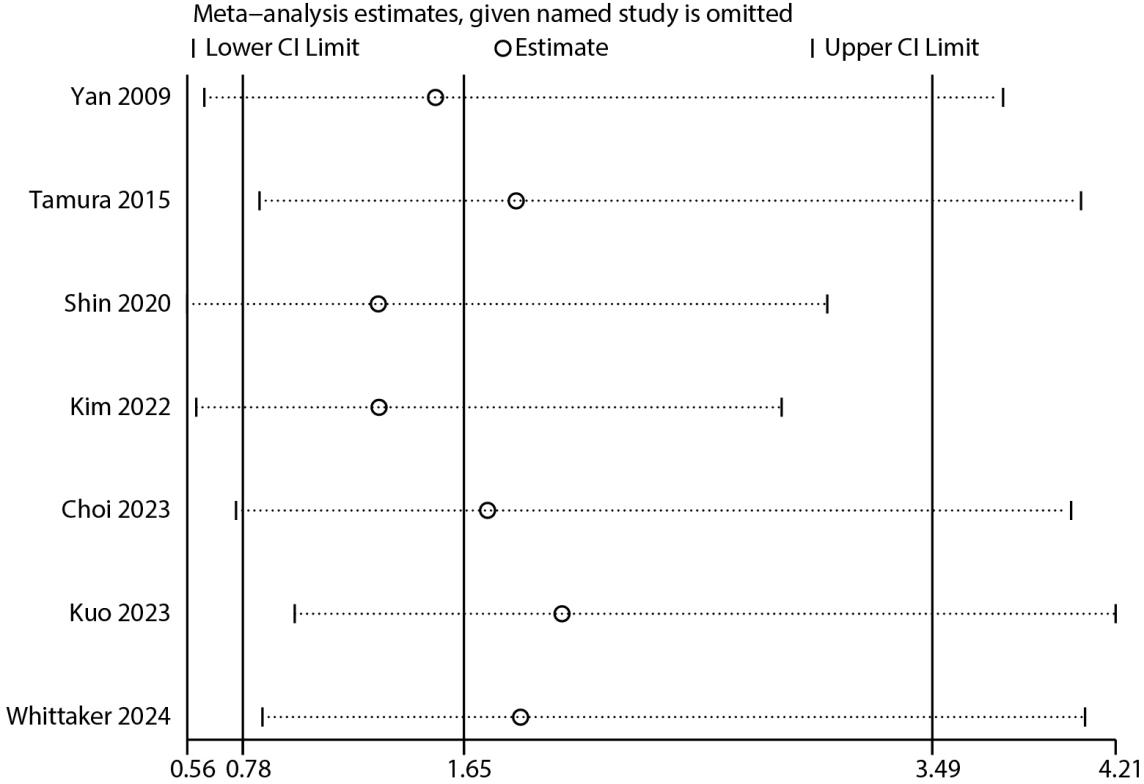


Figure S17. Sensitivity analysis for the association of clinical stage of lung cancer with the risk of CPD in patients with lung cancer


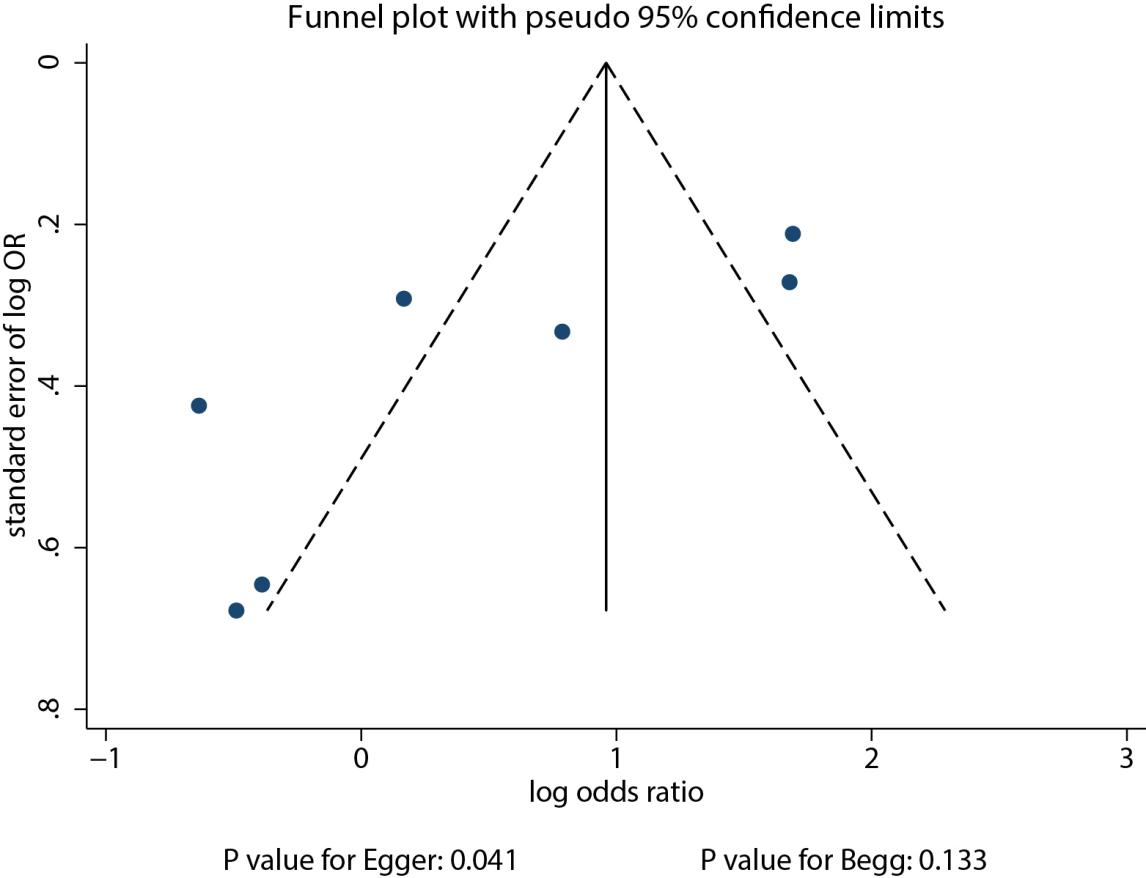


Figure S18. Funnel plot for the association of clinical stage of lung cancer with the risk of CPD in patients with lung cancer
